# Decisional conflict among health care workers regarding the covid 19 vaccine

**DOI:** 10.1192/j.eurpsy.2023.1710

**Published:** 2023-07-19

**Authors:** S. Dhakouani, R. Lansari, N. Hamrouni, A. Larnaout, W. melki

**Affiliations:** 1 psychiatry, Razi hospital, tunis, Tunisia

## Abstract

**Introduction:**

The introduction of the covid 19 vaccine was a long-awaited event. However, many concerns accompanied this vaccine and the decision to be vaccinated was conflicting, especially among the most vulnerable population at that time, the health care workers.

**Objectives:**

Evaluate the decisional conflict among health care workers concerning the launch of the covid19 vaccination campaign.

**Methods:**

This is a descriptive study conducted by a questionnaire posted on social networks using Google Forms targeting groups of health professionals before the launch of the vaccination campaign in Tunisia from January 16, 2021 to March 6, 2021.

We collected sociodemographic data and the attitudes of health care workers about COVID 19 vaccination. We used SURE score to screen the decisional conflict related to COVID 19 vaccine.

**Results:**

Our study included 168 health care workers represented mainly by medical personnel (81% of the respondents). The average age was 34 ±10 years and sex ratio was 0.22.

Sixty percent (60%) of population were hesitant about the COVID 19 vaccine. Scientific sources were consulted by 61% and non-scientific sources were referred to by 19%. Seven percent (7%) did not use any information source.

The SURE decisional conflict score: the mean was 2.26 ± 1.35. The majority of our population (74.4%) had SURE scores in favour of a decisional conflict. We found that decisional conflict was significantly related to information sources. This conflict was highest amoung health care workers who did not use scientific information sources to inquire about the vaccine.

**Conclusions:**

The announcement of the covid 19 vaccination campaigns raised a significant decisional conflict among the health care workers. Screening for decisional conflict among this population is important. Specific interventions to reduce this conflict are recommended by incorporating decision support tools (Decison Aids) and the shared decision making approach.

**Disclosure of Interest:**

None Declared

